# APOBEC3G Variants and Protection against HIV-1 Infection in Burkina Faso

**DOI:** 10.1371/journal.pone.0146386

**Published:** 2016-01-07

**Authors:** Tegwinde Rebeca Compaore, Serge Theophile Soubeiga, Abdoul Karim Ouattara, Dorcas Obiri-Yeboah, Damehan Tchelougou, Mamoudou Maiga, Maleki Assih, Cyrille Bisseye, Didier Bakouan, Issaka Pierre Compaore, Augustine Dembele, Jeremy Martinson, Jacques Simpore

**Affiliations:** 1 Pietro Annigoni Biomolecular Research Centre (CERBA)/ LABIOGENE, University of Ouagadougou, Ouagadougou, Burkina Faso; 2 University of Cape Coast, School of Medical Sciences, Microbiology Department, Cape Coast, Ghana; 3 Laboratory of Molecular and Cellular Biology, University of Sciences and Techniques of Masuku (USTM), Franceville, Gabon; 4 Permanent Secretary against AIDS and Sexually Transmitted Diseases, Ouagadougou, Burkina Faso; 5 Department of Infectious Diseases and Microbiology, Graduate School of Public Health, University of Pittsburgh, Pittsburgh, Pennsylvania, United States of America; Institute of Infection and Global Health, UNITED KINGDOM

## Abstract

Studies on host factors, particularly the APOBEC3G gene, have previously found an association with AIDS progression in some populations and against some HIV-1 strains but not others. Our study had two main objectives: firstly, to screen a population from Burkina Faso for three variants of APOBEC3G previously described, and secondly to analyze the effect of these three variants and their haplotypes on HIV-1 infection with Circulating Recombinant Forms (CRFs) present in Burkina Faso. This case control study involved 708 seropositive and seronegative individuals. Genotyping was done by the TaqMan allelic discrimination method. Minor allele frequencies of rs6001417 (p<0.05), rs8177832 (P<0.05), and rs35228531 (P<0.001) were higher in seronegative subjects. The rs6001417 and rs8177832 SNPs were associated with HIV-1 infection in an additive model (P<0.01). Furthermore the SNP rs35228531 was also associated with HIV-1 infection in a dominant model (P<0.001). Odds ratio analysis of genotypes and alleles of the different APOBEC3G variants showed that there is a strong association between the *minor genetic variants*, genotype of the three SNPs, and HIV-1 status. Haplotype analysis demonstrated that rs6001417, rs8177832, and rs35228531 are in linkage disequilibrium. The haplotype GGT from the rs6001417, rs8177832 and rs35228531 respectively has a protective effect OR = 0.54 [0.43–0.68] with P<0.001. There was also associations between the haplotypes GGC OR = 1.6 [1.1;-2.3] P<0.05, and CGC OR = 5.21 [2.4–11.3] P<0.001, which increase the risk of infection by HIV-1 from almost two (2) to five (5) fold. This study demonstrates an association of rs6001417, rs8177832, and rs35228531 of APOBEC3G with HIV-1 infection in a population from Burkina Faso.

## Introduction

About 35 million people are living with HIV-1 worldwide at the end of 2013, with sub-Saharan Africa paying the highest toll of this infection as it harbors 71% of infected persons worldwide[1]. Phylogenetic studies conducted in Burkina Faso reported that Circulating Recombinant Forms [CRF06_cpx 16/29 (55.17%), CRF02_AG 9/29 (31.03%), A1 2/29 (6.89%), G 1/29 (3.44%), and CRF09_cpx 1/29 (3.44%)] of the HIV-1 subtypes A, G, K, and J strains are predominant [2–7]. These CRFs or inter-subtypes are said to have originated from individuals that were infected by different viruses of two or more subtypes, and are representative of HIV-1 strains in West Africa. Most studies on vaccine development and host-pathogen interaction that have been carried out extensively in the western world, for the development of new antiretroviral therapies are mostly based on subtype B. Recently, efforts have been focused on the host genetic factors that affect disease progression in HIV-1/AIDS [8,9] such as DC-SIGN, CCR5, SAMHD1 and APOBEC3G. In fact, Apolipoprotein B mRNA editing enzyme catalytic polypeptide-like 3G (APOBEC3G) is a potent host defense factor, which interferes with Human Immunodeficiency Virus 1 (HIV-1) [10]. Vif or virion infectivity factor of HIV-1 is able to counteract APOBEC3G antiviral activity by targeting it for degradation in proteasomes [11, 12]. Vif proteins derived from subtypes A, B, CRF01_AE, and CRF_02AG in a study showed non-significant but some-what differential anti-APOBEC3G activity levels based on infectivity profiles while subtype C was highly significant [13, 14]. The APOBEC3G protein is incorporated into newly synthesized viral particles, in the absence of the virion infectivity factor (vif), and hypermutates viral DNA by deamination of cytosine (C) to uracil (U). APOBEC3G polymorphisms, such as rs8177832 (H186R), are thought to be associated with HIV-1 subtype B and C pathogenesis in some ethnic groups [15, 16], though this association is not seen in other populations [17,18]. These previous studies did not take into account the Circulating Recombinant Forms of HIV-1, nor evaluate the effect of APOBEC3G polymorphisms in West African ethnic groups.

In this study we screened a population from Burkina Faso for three variants of APOBEC3G previously described. Additionally, we analyzed the effect of these three variants and their haplotypes on HIV-1 infection with circulating recombinant forms present in Burkina Faso.

## Materials and Methods

### Study populations

The study group consisted of 336 seropositive and 372 seronegative individuals, for a total of 708. Both groups were recruited from the Saint Camille Hospital and the Pietro Annigoni Biomolecular Research Center (CERBA) cohorts and outpatients.

### Sample collection, HIV-1 testing, CD4 T cell counts and plasma viral load

Plasma Samples obtained by venipuncture were tested for HIV-1 infection, using alere determine HIV-1/2 test, and the SD Bioline HIV-1/2 test was used to differentiate between HIV-1 and HIV-2. CD4 cells were enumerated using the BD FACSCount CD4 Reagent kit on a BD FACS COUNT (Becton Dickinson, San Jose). Viral load was determined using the Abbott HIV-1 Real Time Quantitative kit (Promega, USA) on the Abbott *m*2000rt (Abbot Laboratories, Illinois) according to the manufacturer's protocol.

### DNA extraction and genotyping

Genomic DNA was extracted from leucocytes using the "DNA Rapid Salting-Out" technique as described by Miller et *al*. [19]. SNP selection was based on the most relevant APOBEC3G variants, rs8177832 (H186R), rs35228531 and rs6001417 described by [16]. The three markers of APOBEC3G studied were genotyped using standard TaqMan SNP assays (ABI, Foster City, CA) run on the 7500 Fast Real-Time PCR Systems (Life Technologies, California, USA).

### Ethical considerations

Approval for the study was obtained from the National Health Ethic Committee. All study participants or guardians gave their free written and informed consent.

### Statistical analysis

SPSS version 20.0 was used for data analysis. Power-Marker software Version 3.25 was used for the determination of the Hardy-Weinberg equilibrium and the calculation of allele and genotype frequencies. Changes were considered statistically significant at p < 0.05, using the Fisher Exact test. Odds ratio (OR) and confidence intervals (CI) at 95% were calculated to estimate the associations of HIV-1 infection with the rs6001417, H186R, and rs35228531 polymorphisms using Epi Info 7. Logistic regression was performed on all SNPs with statistically significant allele or genotype tests, associating additive, dominant and recessive models with minor allele as the risk allele.

Linkage disequilibrium (LD) was characterized and haplotype frequencies were computed using Power-marker[20], Haploview [21], and PHASE statistical software [22,23]. D’ and r^2^ summary statistics were calculated using Haploview [21]. Power Marker, Haploview and PHASE use the Expectation Maximization (EM) algorithm to determine haplotype frequency distributions in cases of unknown phase. Only haplotypes with a minimum frequency of 5% were considered in the analysis.

## Results

Our study seronegative and seropositive groups were composed respectively of 25% and 24% of males, and females respectively 75% and 76%. The mean age of seropositive group was 30.11+/- 14.85 years, while the mean age of the seronegative group was 27.14+/- 8.84; the median T cell CD4 counts was 302 cells/mm3 (ranging from 4 to >2000 cells/mm3) with a median viral load of 25121 copies/ml (ranging from non-detectable to 2 268 886 copies/ml).

The different allelic and genotypic frequencies of the three APOBEC3G loci for cases and controls are listed respectively in Table 1. Results demonstrated that the three SNPs rs6001417, rs8177832, and rs35228531 genotype, are all in Hardy-Weinberg equilibrium (HWE), as shown in Table 1.

**Table 1:** Association between APOBEC3G polymorphisms and HIV-1 Status analysis.

SNP	Cases (N = 336)	Controls(N = 372)	OR	95% CI	Genotype p-value
**rs6001417**					
**CC**	0.29	0.25	1.22	0.86–1.72	0.28
**CG**	0.54	0.49	0.98	0.73–1.32	0.94
**GG**	0.17	0.26	0.60	0.41–0.88	**<0.01**
**G**^a^	0.443	0.505	0.78	0.63–0.97	**<0.05**
**HWE**^b^	0.072	0.748			
**rs8177832**					
**AA**	0.29	0.26	1.18	0.84–1.67	0.36
**AG**	0.54	0.49	1.21	0.89–1.64	0.24
**GG**	0.17	0.25	0.61	0.41–0.90	**0.01**
**G**^a^	0.438	0.495	0.8	0.64–1	**<0.05**
**HWE**^b^	0.076	0.740			
**rs35228531**					
**CC**	0.45	0.32	1.73	1.26–2.38	**<0.001**
**CT**	0.44	0.48	0.85	0.63–1.16	0.32
**TT**	0.10	0.19	0.48	0.31–0.76	**0.001**
**T**^a^	0.326	0.435	0.63	0.50–0.78	**<0.0001**
**HWE**^b^	0.784	0.678			

^**a**^: Hardy-Weinberg Equilibrium

^**b**^**:** Minor allele

The three SNPs showed significant differences between cases and controls for both allelic and genotypic distributions using a P-value of 0.05, as shown in Table 1.

There is a strong association between the minor genetic variant, genotype of the three SNPs, and HIV-1 status. The rs6001417 G allele displays a protective effect with an OR = 0.78 [95% CI 0.63–0.97, p = 0.022]. The effect seen for the allele, is caused by the effect seen for the homozygous GG genotype with an OR = 0. 61 [95% CI: 0.41–0.09, p = 0.01]. For the rs8177832 SNP, the G allele presents a protective effect, with an OR = 0.8 [95% CI 0.64–1, p = 0.035], due to the effect seen for the homozygous GG genotype OR = 0.61 [95%CI: 0.41–0.09, P = 0.0]. The SNP rs35228531 shows a stronger association with the HIV-1 status. The T allele displays a protective effect (reduces the risk of being infected) with an OR = 0.63 [95% [95% CI: 0.50–0.78, p = 0.00003] caused by the effect observed for the homozygous TT genotype OR = 0.48 [95% CI: 0.31–0.76, p = 0.001]. Conversely, the homozygous CC genotype almost doubles the risk of being infected OR = 1.76 [95% CI: 1.26–2.38; p = 0.0005], the C allele also display the same pattern: OR = 1.60 [95% CI: 1.28–1.99; p = 0.00003], (Table 1). For both rs8177832 and rs6001417, the major allele genotypes AA and CC did not show a significant association (Table 1).

The multinomial logistic regression analysis for HIV-1 infection risk factors showed that in the age groups 10–18, 19–30, and 31–45, there was a significant difference between the repartitions of cases and controls, but these differences did not contribute to the risk of HIV-1 infection. For the genotype CC there was a significant difference of repartition between cases of controls, the multinomial logistic regression analysis also demonstrated that the CC genotype carriers had three (3) times the risk of being infected by HIV-1 OR = 3.38 [95% CI:1.503–7.605], p = 0.003] (Table 2).

**Table 2:** Multinomial logistic regression analysis for HIV-1 risk factors.

	*OR*	*95% CI*	*p-value*
0–9 years old	0.742	0.656–1.476	0.524
10–18 years old	**0.26**	***0*.119–0,570**	**0.001**
19–30 years old	**0.073**	**0.037–0.147**	**0.000**
31–45 years old	**0.353**	**0.173–0.717**	**.004**
Sex	0.985	0.658–1.476	0.942
CC	**3.381**	**1.503–7.605**	**0.003**

OR: Odd ratio; CI: Confidence interval; CC: genotype of the variant rs35228531

Our results seemed to show that for the association between 3 single loci and HIV-1 status, based on additive, dominant and recessive models, the most significant genotype effect for rs6001417 was an additive model OR = 1.68 [95% CI 1.78–2.40, p = 0. 005]. For the H186R SNP rs8177832, the additive model A/A (referent) vs A/G, G/G OR = 1.651 [95% CI: 1.15–2. 37, p = 0. 008] was the most significant genotype, and finally the most significant genotype effect for rs35228531 was the dominant model (CC vs. CT & TT), OR = 1.73 [95% CI 1.26–2.38, p = 0.0005] (Table 3). The high degree of linkage disequilibrium (LD) seen between these loci prevents us from determining whether each of the 3 loci has an independent effect on HIV-1 status, however, so we further analyzed our data by haplotype construction.

**Table 3:** Association between 3 single loci and HIV-1 Status, Based on Additive, Dominant and Recessive Models.

Markers	Models	OR	95% CI	p-Value
**rs6001417**	C/C (referent), C/G, G/G, additive	1.68	1.78–2.40	**<0.01**
**rs6001417**	C/C *vs*. C/G & G/G, dominant	1.22	0.86–1.72	0.28
**rs6001417**	T/T *vs*. C/C & C/T, recessive	0.60	0.41–0.88	**<0.01**
**rs8177832**	A/A (referent), A/G, G/G additive	1.65	1.15–2.37	**<0.01**
**rs8177832**	A/A *vs*. A/G &G/G, dominant	1.18	0.84–1.67	0.36
**rs8177832**	G/G *vs*. A/A &A/G, recessive	0.61	0.41–0.90	**0.01**
**rs35228531**	C/C (referent), C/T, TT, additive	1.11	0.68–1.78	0.7663
**rs35228531**	C/C *vs*. C/T & T/T, dominant	1.73	1.26–2.38	**<0.001**
**rs35228531**	T/T *vs*. C/C & C/T, recessive	0.48	0.31–0.76	**0.001**

**Fig 1:**
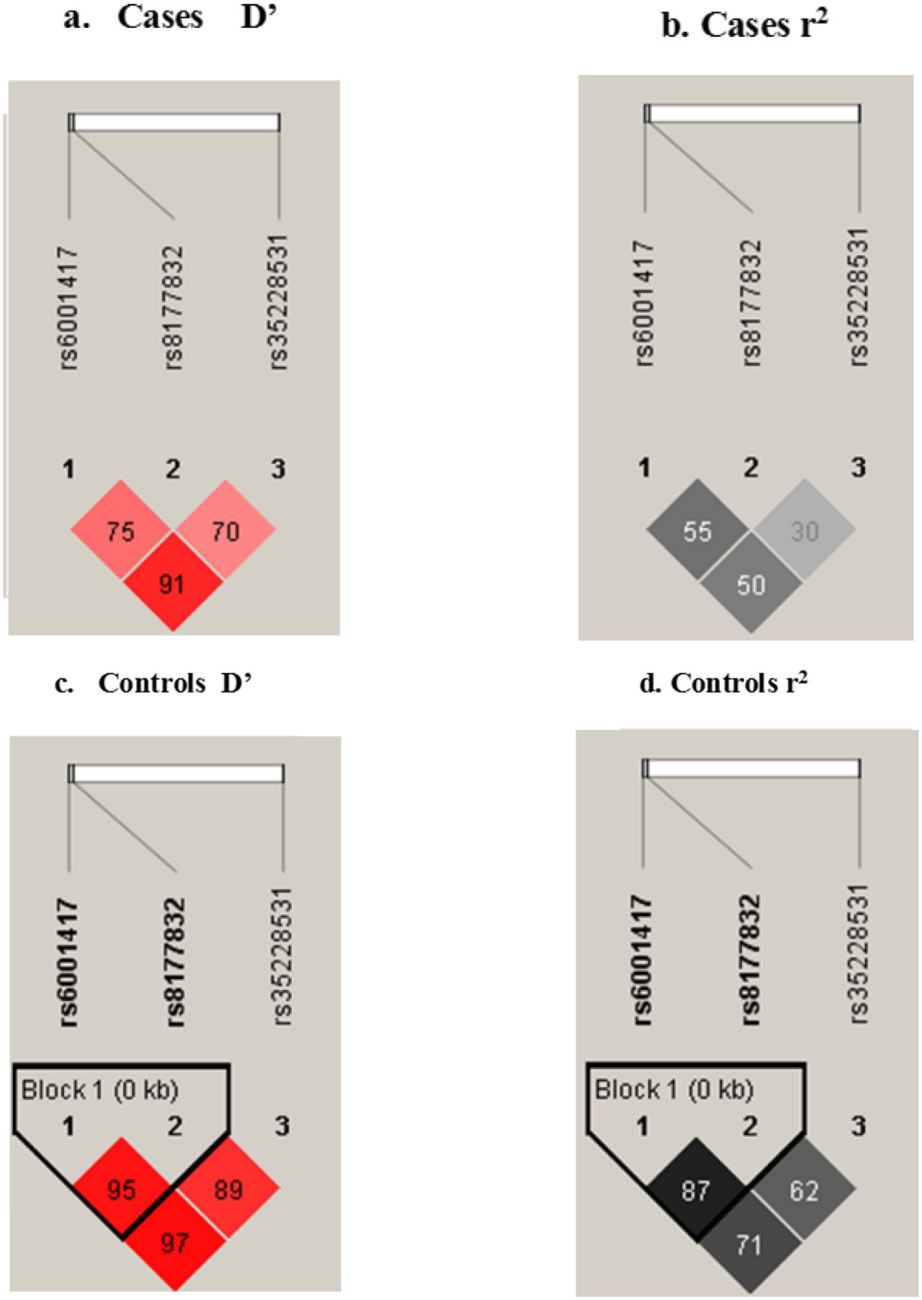
Haploview APOBEC3G linkage disequilibrium plots for case and controls. The figures are oriented 5’ to 3’, right to left, relative to the gene orientation on the minus strand. Fig 1a represents the LD plot of the case pairwise D’ between markers, and Fig 1b shows the LD plot of the control pairwise D’ between markers. Strong LD is indicated by red, while pink indicate uninformative values. LD blocks were created with the default algorithm in the Haploview software (version 4.1) that creates 95% confidence bounds. D’ was considered strong where 95% of the comparisons made are informative.

Haplotype trend regression analysis was followed by LD and r^2^ analysis within the APOBEC3G gene using Haploview software (Fig 1). The linkage disequilibrium between rs6001417 and rs35228531 is very strong in both cases (D’ = 0.91) and controls (D’ = 0.97); their respective r^2^ values were 0.506 for cases and 0.715 for controls. There is also a strong linkage disequilibrium between rs6001417 and rs8177832 for both cases (D’ = 0.755; r^2^ = 0.556) and controls (D’ = 0.955, r^2^ = 0.874). LD for rs8177832 and rs35228531 was weaker for both cases (D’ = 0.7), and controls (D’ = 0.894) compared to the latter (Fig 1).

**Fig 2:**
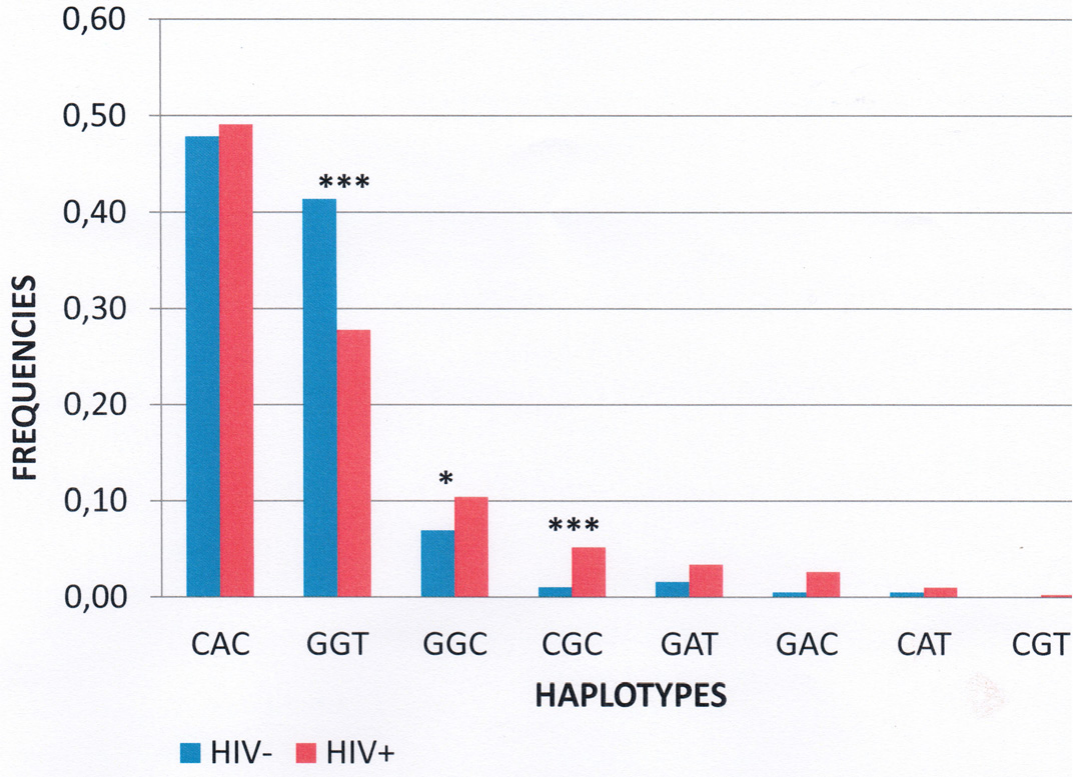
Distribution of the cases and controls haplotypes.

Haplotype frequencies were compared between cases and controls for the APOBEC3G gene, using all 3 SNPs (Table 4). The p-value for the overall case-control comparison was 0.01, indicating that the HIV-1+ and HIV-1- sets have significantly different haplotype distributions. This difference is apparently due to the higher frequency of the GGT haplotype in the HIV-1 negative samples (41%) compared with the HIV-1 positive samples (28%) (Fig 2). For the GGT genotype, there was a protective effect against HIV-1 infection with OR = 0.56 [95%CI; 0.45–0.71, p<0.001] (Table 4). There were also associations between the genotypes GGC, OR = 1.6 [95%CI 1.1–2.3, p<0.05], and CGC OR = 5.21 [95%CI: 2.4; 11.3, p<0.001], which increase the risk of infection from almost two (2) to five (5) times by HIV-1 (Table 4).

**Table 4:** Cases and controls Haplotypes.

Haplotype	Cases	Controls	OR	CI	p-Value
**CAC**	330	356	1,05	0,85; 1,29	0.67
**CGC**	35	8	**5,21**	2.4; 11.3	**<0.001**
**GGC**	70	52	**1.6**	1.1; 2.3	**<0.05**
**GGT**	187	308	**0.56**	0.45; 0.71	**<0.001**

The distribution of the case and control haplotypes is shown in Fig 2. The loci are shown in the order: rs6001417- rs8177832- rs35228531. This represents the frequencies of the different haplotypes present among cases (blue) and controls (orange). The differences were statistically significant for the haplotypes shown as “*” (p<0.05), and “***” (p<0.001).

## Discussion

We investigated the distribution of three genetic variations within the APOBEC3G gene in a population from Burkina Faso, where the HIV-1 Circulating Recombinant Forms CRF06_cpx and CRF02_AG dominate [2]. We studied the APOBEC3G gene regardless of the fact that Vif or virion infectivity factor of HIV-1 is able to counteract APOBEC3G antiviral activity by targeting it for degradation in proteasomes [11, 12]. Although HIV-1/AIDS affects Sub-Saharan Africa the most, not many studies have been conducted on the definitions of AIDS restriction genes on native populations, in which genotype frequencies depend on ethnic background [15], [24]. In this study, we describe the frequency of 3 SNP genotypes and their haplotypes. There have been very few studies on rs6001417 and rs35228531 since their identification by Reddy et al.

The H186R mutation of APOBEC3G has been reported in the literature to be associated with an accelerating effect on disease progression in African Americans infected with HIV-1 subtype B at a frequency of 0.37 [15]. Furthermore, the latter effect was also reported in a South African cohort infected with HIV-1 subtype C carrying the H186R mutation with a frequency of 0.307 [16]. This polymorphism is also associated with rapid disease progression in a population of HIV-1 subtype B infected children. In our study, this mutation occurred at a frequency of 43.8% in a population infected with HIV-1 circulating recombinant forms CRF06_cpx and CRF02_AG. The genotyping results showed that all 3 loci followed the Hardy Weinberg Equilibrium.

Our statistical analysis revealed that the 3 loci tested did not show an association with CD4 T cell count or viral load (Data not shown). This finding in our study population of Burkina Faso implies that the host genetic variations play a greater role in the hyper-mutations than viral genetic variation.

The results suggest that the minor alleles G, T and G are protective against infection in homozygotes for rs6001417, rs8177832, and rs35228531 respectively, with OR = 0.78 [95% CI: 0.63–0, 97, p = 0.022], OR = 0.61 [95% CI: 0. 41–0. 09, p = 0. 01], and OR = 0.63; [95% CI: 0.50–0.78, p = 0.00003], but these results may be due to the high linkage disequilibrium that exists between them.

The variants of rs35228531 reduced greatly the risk of being infected with HIV-1 for the carriers of the minor allele T in the homozygous state with an OR: 0.63 [95% CI: 0.50–0.78, p = 0.00003] and an almost doubled risk of HIV-1 infection when the wild type allele C is present in homozygotes, OR = 1.76 [95% CI: 1.26–2.38; p = 0.0005]. Furthermore, the genotype CC after a multinomial logistic regression showed a tripled (3) risk of infection to HIV-1 (OR = 3.381; [95%CI: 1.503–7.605), p = 0.003]. The action of rs35228531 on APOBEC3G activity is still not well defined. It is an extragenic mutation located near the 3’ region of the gene, and might therefore interfere with transcriptional and post-transcriptional regulation of the gene and increase its antiviral activity. In fact, SNPs may influence promoter activity (gene expression) and thus modulate the risk of infection [25, 26].

Linkage disequilibrium was strong in all 3 pairs of loci in the Burkinabe APOBEC3G dataset, which causes a non-independence of allele segregation. Physical proximity (about 6kb) of these 3 loci on the gene region of APOBEC3G could explain the strong linkage in all alleles. Linkage disequilibrium (LD) plots characterizing haplotype haplotypes blocks in APOBEC3G are shown in Fig 1a and 1b. The association patterns differ in cases and controls, as shown in Fig 1, a result that can be explained by the fact that the cases showed lower levels of LD compared to controls.

We observed evidence for haplotype-specific associations in the HIV-1 infected group compared to control subjects. In fact, for the GGT genotype there was a protective effect against HIV-1 infection with OR = 0.56 [95% CI: 0.45–0.71; p<0.001] (Table 4). There were also associations between the genotypes GGC (OR = 1.6 [95% CI: 1.1–2.3; p<0.05]) and CGC (OR = 5.21 [2.4–11.3, p<0.001]), increasing the risk of infection from almost two (2) to five (5) times by HIV-1 (Table 4). These results can be explained by the strong LD that exists between the different loci. They can also be justified by the fact that rs6001417, rs8177832 and rs35228531 are in linkage disequilibrium [16], which is confirmed in our study (Fig 1), therefore the haplotypes rs8177832, rs35228531 and rs6001417 will have the same effect.

This study shows that the variants rs8177832, rs3522851 and rs6001417 are in linkage disequilibrium. Furthermore, this study demonstrates that APOBEC3G is a susceptibility gene for HIV-1 infection in a West African population from Burkina Faso. To our knowledge, this is the first study investigating the role of APOBEC3G variants on an HIV-1 CRF06_cpx and CRF02_AG infection. The effect of APOBEC3G polymorphisms in our study population suggests that APOBEC3G sequence variation plays a role at a biological level in the interaction of HIV-1 and the host. The precise role of APOBEC3G and its variants in HIV-1 susceptibility needs to be elucidated in further studies.
